# Explant Type, Culture System, 6-Benzyladenine, Meta-Topolin and Encapsulation Affect Indirect Somatic Embryogenesis and Regeneration in *Carica papaya* L.

**DOI:** 10.3389/fpls.2018.01769

**Published:** 2018-12-04

**Authors:** Paúl Solórzano-Cascante, Neiva Sánchez-Chiang, Víctor M. Jiménez

**Affiliations:** ^1^Centro para Investigaciones en Granos y Semillas, Universidad de Costa Rica, San Pedro, Costa Rica; ^2^Instituto de Investigaciones Agrícolas, Universidad de Costa Rica, San Pedro, Costa Rica; ^3^Food Security Center, University of Hohenheim, Stuttgart, Germany

**Keywords:** somatic embryogenesis, meta-topolin, logistic regression, culture systems, synthetic seeds

## Abstract

A protocol to propagate papaya hybrid plants through indirect somatic embryogenesis was developed considering the effect of explant type, culture system, particular cytokinins and encapsulation, in different stages of the process. Optimal 2,4-dichlorophenoxyacetic acid (2,4-D) concentrations for non-embryogenic callus formation ranged between 9.0 and 27.1 μM in half-cut seeds, while higher concentrations were harmful. Non-embryogenic callus was also obtained with 22–158 μM 2,4-D from hypocotyl segments. Callus with embryogenic structures was only obtained in half-cut seeds cultured in the darkness on half-strength Murashige and Skoog culture medium supplemented with 2,4-D, while hypocotyl segments and isolated zygotic embryos failed to produce this type of callus regardless of the 2,4-D and sucrose (30 and 70 g l^-1^) concentrations tested in this study. Both, embryogenic callus development and quantity of somatic embryos formed per embryogenic callus, which ranged between 11 and 31 units after 14 months, required 2,4-D, but without any effect of the concentration. Histological studies confirmed the multicellular origin of the somatic embryos. In further steps, liquid medium induced over four times more somatic embryos than agar-gelled medium and showed significantly higher production of globular somatic embryos (85 vs. 57%). Both, 6-benzyladenine (BA) and meta-topolin (Mtop) stimulated sprouting (40–45%) of the somatic embryos (development of shoots only) in concentrations of up to 2.7 and 10 μM, respectively. Sprouting probability showed a 2nd order polynomial trend despite the range of concentration used for each cytokinin. This is the first report about the positive effect of Mtop on the apical shoot development of *Carica papaya* somatic embryos known to the authors. Radicle growth was observed in 5% or less of the cultivated embryos, regardless of the BA concentration. Finally, all encapsulation conditions tested (2.5, 3.5, and 4.5% sodium alginate, combined with 50 and 100 mM CaCl_2_) reduced sprouting of somatic embryos when compared to the non-encapsulated ones, whereas capsule hardness showed low correlation with embryo sprouting. Embryos were further cultivated until they became plantlets approximately 5 cm long. They were acclimatized and afterward planted in the field, where they flowered and produced fruit.

## Introduction

*Carica papaya* L. is the only species in its genus and, due to the fruit nutritional value, it is of great interest for human nutrition. Papaya plants are trioecious, semi-perennial and have semi herbaceous stems (Jiménez et al., 2014). Incipient sexual chromosomes control sex determination in papaya and there are no vegetative traits that allow determining the sex of young plants before flowering (Aryal and Ming, 2014; Jiménez et al., 2014).

Papaya breeding programs through conventional techniques have been developed to improve the quality of the fruit (Rimberia et al., 2018). One of this quality traits is the shape of the fruit, since, commercially, papaya fruits harvested from hermaphrodite plants are preferred over those from female plants, due to their reduced internal cavity and elongated form (Chan-Tai et al., 2003; Talavera-May et al., 2007; Mora-Newcomer and Bogantes-Arias, 2012). Moreover, papaya is an allogamous species with the disadvantage that parents of some commercial hybrids produce few seeds and the germination percentages are low, making its high-scale propagation difficult (Bhattacharya and Khuspe, 2001; Webster et al., 2016). Therefore, somatic embryogenesis offers the possibility of propagating only hermaphrodite plants (after screening) of selected genotypes at high rates.

There is increasing evidence that somatic embryogenesis in papaya, like in most plants, is genotype-dependent (Dhekney et al., 2016), with some genotypes responding better than others to particular conditions (explant type, medium supplements, culture conditions, etc.). Up to date, most success has been achieved with some Hawaiian cultivars (e.g., “Solo” and “Sunrise”) (Chen et al., 1987; Castillo et al., 1998; Cai et al., 1999), while others still lack behind.

Development of somatic embryogenesis has been divided into two phases, induction and expression (Jiménez, 2005). Auxins play an important role in cell transition from somatic to embryogenic states (induction) in many plants, with 2,4-dichlorophenoxyacetic acid (2,4-D) as the most used compound for this purpose, and papaya is not an exception (Fitch and Manshardt, 1990; Mishra et al., 2007; Posada-Pérez et al., 2007). On the other side, in order to promote the germination of papaya somatic embryos, in the expression phase, cytokinins, such as 6-benzyladenine (BA) and kinetin, at concentrations of 0.9 and 18.6 μM, respectively, have been successfully used (Mishra et al., 2007; Posada-Pérez et al., 2007). Use of meta-topolin (Mtop), a hydroxylated BA analog reported to have less negative carryover effects (residual effects of the compound beyond the time period in which it was present) during *ex vitro* acclimatization of several plant species (Werbrouck et al., 1996), has not been reported in papaya somatic embryo germination and might deserve attention.

*Ex vitro* acclimatization is the final step that needs to be set up for any successful micropropagation protocol. In many cases, this is a major bottleneck and needs to be approached carefully, considering carryover effects (e.g., irregular stomata functionality) from the *in vitro* phase and adequate design of the acclimatization conditions (Shahzad et al., 2017). To facilitate this phase, somatic embryos, as well as other propagules, can be encapsulated in calcium alginate or other matrixes in order to protect and enable their easier handling (Rai et al., 2009). Capsule hardness, resulting from the concentration of alginate and calcium, has an influence on embryo development and germination (reviewed by Gantait et al., 2015), and needs to be experimentally assessed case by case (e.g., Haque and Ghosh, 2016; Raju et al., 2016; Prakash et al., 2018).

One of the used models for the analysis of binary data (e.g., number of responsive explants, germinated somatic embryos, etc.) is the logistic regression. This model denotes the success probability for a specific binary variable to occur according to the value of the independent and quantitative factor (Agresti, 2007). Logistic regression has been used to analyze the rooting response of shoots of several *Eucalyptus dunnii* cultivars (Oberschelp et al., 2015; Oberschelp and Gonçalves, 2016), as well as for the formation of callus with embryogenic structures from immature embryos of *Pinus radiata* (García-Mendiguren et al., 2016).

The main objectives of the present work are to evaluate the embryogenic potential of several explants of a papaya hybrid, to induce development of embryogenic structures, to germinate them, emphasizing on the role that Mtop can play, and to acclimatize the obtained plantlets. In addition, the effect of the components concentration on the capsule hardness and embryo protrusion during encapsulation was assessed.

## Materials and Methods

### Plant Material

Three explant types were assessed to induce embryogenic callus: hypocotyls, half-cut seeds, and isolated zygotic embryos. Papaya seeds of a F1 hybrid resulting from crossings between homogeneous lines, one originated from the Hawaiian Papaya Breeding Program (“Solo”-type) and the other a Costa Rican landrace (Papaya Breeding Program of the University of Costa Rica), were used throughout the experiments. Seeds employed for germinating plantlets, later used for dissecting the hypocotyls, were obtained from the Fabio Baudrit Moreno Experimental Station of the University of Costa Rica, located in Alajuela (latitude: 10.008025, longitude: -84.265897). The rest of the seeds used in this study were produced at “Los Diamantes” Experimental Station of the Costa Rican Ministry of Agriculture and Husbandry, located in Guapiles, Limón (latitude: 10.2594, longitude: -83.7715). In every case, seeds were aseptically extracted from physiologically mature fruits and dried at room temperature for a week under constant air flow in a laminar flow cabinet. The dry seeds were stored in sterile glass containers inside a desiccator at 20–30% relative humidity and 25°C until the establishment of the experiments.

Half-cut seeds were prepared by simply dividing the dry seeds with a scalpel and forceps and placing the cut surface in contact with the culture medium. The isolated zygotic embryos were extracted under a stereoscopic microscope, using a scalpel and forceps as well. Hypocotyl sections (2 mm in diameter) were excised from 23-day-old plantlets, aseptically germinated and grown on water/agar (8.0 g l^-1^) under light conditions, and placed horizontally on the culture medium.

### Induction and Characterization of Callus

The semi-solid basal medium was composed by half the concentration of Murashige and Skoog (1962) salts, supplemented with thiamine⋅HCl (0.1 mg l^-1^), pyridoxine⋅HCl (0.5 mg l^-1^), nicotinic acid (0.5 mg l^-1^), glycine (2 mg l^-1^), myo-inositol (100 mg l^-1^), L-glutamine (400 mg l^-1^), sucrose (30 g l^-1^) and Phytagel (2.8 g l^-1^) (Sigma, St. Louis, MO, United States). Half-cut seeds and isolated zygotic embryos were cultured on this induction basal medium, supplemented with 2,4-D (9.0, 18.0, 27.1, 36.2, or 45.2 μM) (*n* = 4). Besides, the effect of sucrose concentration (30 and 70 g l^-1^) was tested on half-cut seeds cultured with 9.0 μM 2,4-D (*n* = 8). In the case of hypocotyl sections, higher concentrations of 2,4-D were also tested (22.6, 67.8, 113.1, and 158.3 μM), in combination with the sucrose concentrations mentioned above (*n* = 10). In every case, 25 ml of autoclaved medium (1.05 kg cm^-2^; 30 min) was poured into Petri dishes (90 mm diameter). The pH of the culture media was adjusted to 5.80 (±0.01) with KOH. Every culture mentioned above was maintained under dark conditions, and at 24–25°C and 48% relative humidity, during the entire period of evaluation. After 8 and 14 weeks of culture, the number of explants with callus (with and without embryogenic structures) and the production of somatic embryos was evaluated. Each repetition consisted of one Petri dish with four explants. To increase the amount of callus with embryogenic structures, subcultures were done every 2 months to the corresponding culture medium.

Histological analyses were performed on somatic embryos after 8 months of culture and on embryogenic callus developed from half-cut seeds on 9.0 μM 2,4-D according to Prophet et al. (1994), Leong (2004), and Buesa (2007), as summarized below. Tissues were immersed in FAA (formalin-acetic acid-alcohol) solution for 72 h. FAA was removed by rinsing the tissues with distilled water for 2 h. Dehydration of the tissues was conducted through a gradient of ethanol dilutions (50 to 100% v/v). Finally, tissues were immersed in absolute ethanol and xylene (1:1 v/v) for 2 h. For paraffin infiltration the tissues were immersed for 30 min in xylol: liquid paraffin (1:1 v/v) followed by liquid paraffin (twice), both solutions were at 65°C. Tissues were subsequently sliced 5–10 μm thick with a rotary microtome (American Optical 820, Buffalo, NY, United States). Paraffin was removed from the callus tissues using xylol and ethanol dilutions. Tissues were stained with toluidine blue (1% v/v). Slides were examined with an inverted microscope OLYMPUS IX51 (Olympus, Tokyo, Japan). Images of the most representative preparations were captured with an Olympus DP71 digital camera (Olympus, Tokyo, Japan). GIMP 2.10.6 was used to adjust contrast and brightness.

### Multiplication and Germination of Papaya Somatic Embryos

To assess the effect of using liquid culture systems over the multiplication rate of papaya somatic embryos, embryogenic calluses, derived from half-cut seeds, which were induced with the different concentrations of 2,4-D used in the previous experiment, were mixed after 6 months of culture. From this callus pool, 0.5 g per repetition was placed on semi-solid or in liquid callus formation basal culture medium supplemented with 9.0 μM 2,4-D. The liquid cultures (25 ml) in 50 ml Erlenmeyer flasks, were agitated at 120 rpm in an orbital shaker (Hotech^®^, San Chung, Taiwan). Culture conditions were the same as described above. Subcultures were conducted every month and 15 days for the semi-solid and liquid cultures, respectively. After 2 months of culture, production of somatic embryos, fresh weight and number of cotyledonary and hearth/torpedo somatic embryos were visually evaluated. For this experiment, five repetitions were established for each culture system. The somatic embryos developed in this experiment were later used for the germination assays.

The effect of different concentrations of BA (0.0, 0.9, 1.8, 2.7, or 3.6 μM) and Mtop (0.0, 5.0, 10.0, 15.0, or 20.0 μM) added to the culture medium over further development (germination) of somatic embryos was evaluated in two additional experiments. For the first cytokinin, somatic embryos cultivated for 2-months on a semi-solid culture medium with 9 μM 2,4-D were used, while, for Mtop it was 12-month old-exposed cultures. For these experiments, the basal medium was composed of MS salts, the same concentration of vitamins and sucrose as described above, and agar (9.0 g l^-1^) (Riedel-de Haën, Seelze, Hannover). Culture conditions were the same as described above, but under a 12 h photoperiod (46 μmol m^-2^ s^-1^, Sylvania Supersaver Cool White, 32 W, F48T12/CW/SS).

For the BA experiments, five Petri dishes with four clusters of somatic embryos (5–10 somatic embryos/cluster) at the cotyledonary stage (0.5–1.0 mm in length) were established. The Mtop experiment was conducted with four Petri dishes with four callus sections with approximately 20 somatic embryos each one. Explants were subcultured after 1 and 2 months of culture, when the number of germinated (with evident radicle and epicotyl growth) or sprouted (only epicotyl growth was observed) somatic embryos was evaluated.

### Encapsulation of Papaya Somatic Embryos

To evaluate the effect of encapsulation over germination of somatic embryos, encapsulated and non-encapsulated somatic embryos were induced to germinate. To soften the calcium alginate capsule, part of the encapsulated embryos were subjected to a treatment with KNO_3_ as described below.

For encapsulation, 8 month-old somatic embryos at the cotyledonary stage, developed on the semi-solid culture system supplemented with 9.0 μM 2,4-D, were used. The effect of the concentration of medium-viscosity (80 mPa s) sodium alginate (2.5, 3.5, 4.5% p/v) and CaCl_2_ (50 and 100 mM) over the hardness of the surrounding capsule and on the sprouting of the encapsulated somatic embryos was evaluated. The sodium alginate solutions were supplemented with half the concentration of MS salts, except for Ca (to avoid gelification of the solution), sucrose (30 g l^-1^) and BA (1.8 μM). The pH of the solutions was adjusted to 5.8 (±0.01) and then autoclaved (121°C, 1.05 kg cm^-2^ for 30 min). The encapsulation procedure was based on Castillo et al. (1998) modified as follows. Cotyledonary somatic embryos (0.5–1.0 mm in length) were dipped in the solutions of sodium alginate, suctioned individually with 200 μl of this solution using a pipette (2.5 mm tip internal diameter) and dispensed in the CaCl_2_ solutions. After 30 min the capsules were removed from the CaCl_2_ solutions and rinsed three times with sterile distilled water.

As mentioned above, the capsule of part of the encapsulated somatic embryos was softened with KNO_3_. For that, encapsulated embryos were immersed, for 30 min, in an autoclaved KNO_3_ solution (200 mM). They were then rinsed three times with sterile distilled water before culture.

Non-encapsulated and encapsulated (both with and without KNO_3_ treatment) somatic embryos were cultured on the same basal medium used for the germination experiment supplemented with BA (1.8 μM). The somatic embryos were cultured under light conditions as specified above. Five somatic embryos (encapsulated or not) were placed per Petri dish and each treatment consisted of five Petri dishes (repetitions). After 1 month of culture, the percentage of sprouted and oxidized somatic embryos (i.e., embryos showing tissue browning) was evaluated. This latter variable was analyzed to assess the oxidation stress associated with the encapsulation matrix.

In addition, hardness of calcium alginate capsules resulting from the combination of the sodium alginate and CaCl_2_ concentrations detailed above (without a somatic embryo inside) was evaluated with a digital texturometer (TA-TX Plus Texture Analyzer, Stable Micro Systems LTD., Godalming, United Kingdom) equipped with a cylindrical tip (3 mm diameter), which penetrated 2.5 mm inside the capsules. The hardness of the capsules was measured as the strength of rupture, which was determined as the area under the curve since the beginning of the penetration of the tip and until the capsules were broken.

### *Ex vitro* Acclimatization of Plantlets

Plantlets originated from somatic embryos that showed a normal radicle growth after the germination phase, specifically from the BA germination experiment, were subcultured into the same culture medium, in this case without cytokinins and under the conditions used in the germination phase until they reached 5–6 cm in height and a well-developed root system. Monthly subculturing of the plantlets was conducted for approximately 6 months. Subsequently, plantlets were cultured in a mixture of peat moss and perlite (1:1 v/v) and kept under a transparent plastic micro tunnel inside a greenhouse for approximately 3 months. Plants were then transplanted to a field plot at “Los Diamantes” Experimental Station mentioned above, a region characterized by its tropical humid conditions, at a density of 1320 plants ha^-1^. Plants were nourished with 130 and 750 g of chemical fertilizer composed by 10:30:10% w/w (N – P_2_O_5_ – K_2_O) and 18-5-15-6-2% w/w (N – P_2_O_5_ – K_2_O – MgO – B_2_O_3_), respectively, in eight applications throughout the culture period.

### Statistical Analysis

All variables evaluated in the experiments using hypocotyl segments, as well as the variables “callus formation” and “oxidation of somatic embryos” assessed during the germination experiments, were analyzed using chi-square. The number of explants with embryogenic callus from the half-cut seeds using a range of 2,4-D from 9 to 45 μM was analyzed as a logistic regression with a second-grade polynomial approach. The number of embryos formed per explant during the induction stage was analyzed with chi-square. All the variables evaluated at the multiplication of the somatic embryos using semi-solid and liquid culture systems were evaluated with one-way analysis of variance (ANOVA). A logarithm (base 10) transformation was applied to all data from that experiment due to lack of normality and variance homoscedasticity. Sprouting of somatic embryos was analyzed as a logistic regression with a second level polynomial and with a chi-square analysis. Sprouting and oxidizing percentage of encapsulated embryos and hardness of the capsules were analyzed with a factorial ANOVA followed by a mean comparison with Tukey’s test. Due to lack of normality, a square root transformation was applied to sprouting and oxidizing percentages. To compare the response obtained from the encapsulated and non-encapsulated somatic embryos, both variables were analyzed by chi-square. A completely randomized design was used for all the experiments mentioned above. Analyses of variance, chi-square analyses, and logistic regression analyses were conducted with R studio (R Studio Team, 2016) and R software (R Development Core Team, 2008).

## Results

### Formation and Characterization of Callus

Development of callus (non-embryogenic) occurred in 91–99% of the hypocotyl segments cultured with 2,4-D (22–158 μM) regardless the sucrose concentration tested (30 or 70 g l^-1^) after 8 weeks of culture (Table 1). Concentrations higher than 67.8 μM 2,4-D in combination with 70 g l^-1^sucrose induced 5–8% less non-embryogenic callus in the hypocotyl segments compared to the rest of the treatments. Isolated zygotic embryos cultured on the callus formation medium supplemented with 2,4-D (9–45 μM) did neither form callus nor somatic embryos in any of the established treatments during the entire evaluation period.

**Table 1:** Callus formation on papaya hypocotyl sections after 8 weeks of culture on half-strength MS medium with different concentrations of sucrose and 2,4-D.

	Sucrose (g l^-1^)	
2,4-D (μM)	30	70
22.6	99 a	99 a
67.8	97 a	98 a
113.1	99 a	91 b
158.3	96 a	91 b


*Letters indicate significant differences between treatments based on Chi-square analysis (*p* < 0.05).*

**FIGURE 1:**
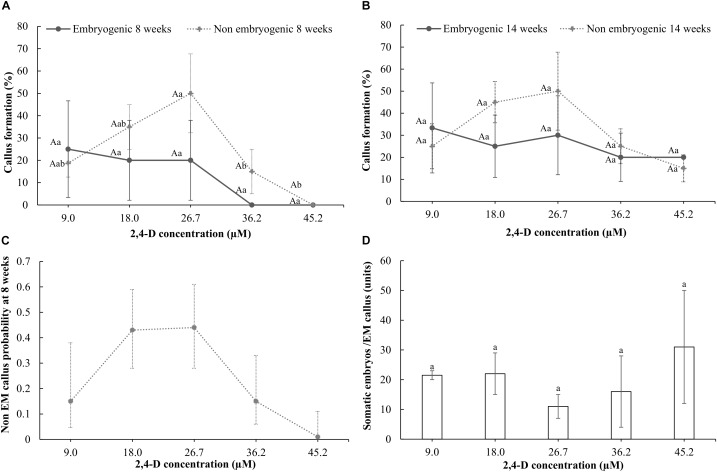
Response of half-cut seeds to different concentrations of 2,4-D (9.0, 18.0, 27.1, 36.2, or 45.2 μM) after 8 **(A)** and 14 weeks of culture **(B)**. Probability of formation of callus with embryogenic structures in half-cut papaya seeds cultured for 8 weeks **(C)**; Wald *t*-test: *X^2^* = 8.8, *p*(>*X*^2^) = 0.0031. Estimated probability equation: P(y) = 1/(1+e^(4.136-0.4x+0.00921x^2^)^); *p-*value = 9.744555e^-06^; and the quantity of somatic embryos formed per each embryogenic (EM) callus after 14 weeks of culture **(D)**. Vertical bars correspond to the standard deviation of the means **(A,B,D)** or of the calculated probabilities **(C)**. Uppercase letters denote significant differences between weeks of culture for the same treatment and type of callus. Lowercase letters denote significant differences between treatments for each week of evaluation and type of callus.

After 8 weeks of culture with 9 μM 2,4-D, half-cut seeds cultured with 30 g l^-1^ sucrose showed higher production (65%) of non-embryogenic callus than when 70 g l^-1^ sucrose was used (35%) (*p* = 0.013). Therefore, 30 g l^-1^ sucrose was used afterward. Development of non-embryogenic callus from half-cut seeds after 8 weeks of culture showed a similar response in the range from 9.0 to 26.7 μM 2,4-D. Concentrations higher than 26.7 μM 2,4-D showed 40–50% less non-embryogenic callus formation (Figure 1A). Logistic regression analysis of non-embryogenic callus formation after 8 weeks of culture showed a significant effect of 2,4-D concentration [*X*^2^= 8.8, *p*(>*X*^2^) = 0.0031] and of the model fitting (*p*-value = 9.744555e^-06^) where the predicted probabilities showed a similar tendency with the results obtained (Figures 1A,C). After 14 weeks of culture, non-embryogenic callus formation on the half-cut seeds ranged from 15 to 50% in all concentrations tested [*p* > 0.05 for Chi square test and *p*(>*X*^2^) > 0.05 for the logistic regression analysis]. After 14 weeks of culture, rate of non-embryogenic callus formation did not increase at any specific 2,4-D concentration compared with the response at 8 weeks of culture (Figures 1A,B).

Regarding development of embryogenic structures, they were not observed originating from the hypocotyl segments or from the callus formed from them, regardless the 2,4-D or sucrose concentrations used after a culture period of 16 weeks. Moreover, the concentration of sucrose neither significantly (*p* = 0.608) affected the embryogenic callus formation of the half-cut seeds, which ranged from 2.5 to 7.5%, after 8 weeks of culture on a culture medium with 9.0 μM 2,4-D. In addition, the concentration of 2,4-D in the culture medium with 30 g l^-1^ sucrose did not affect the embryogenic callus formation rate on the half-cut seeds after either 8 or 14 weeks of culture, ranging between 20 and 30% after 14 weeks (Figures 1A,B). Finally, the number of somatic embryos formed in the embryogenic callus ranged between 11 and 31 somatic embryos after 14 weeks of culture without any significant effect of the 2,4-D concentration used (*p* > 0.05) (Figure 1D).

**FIGURE 2:**
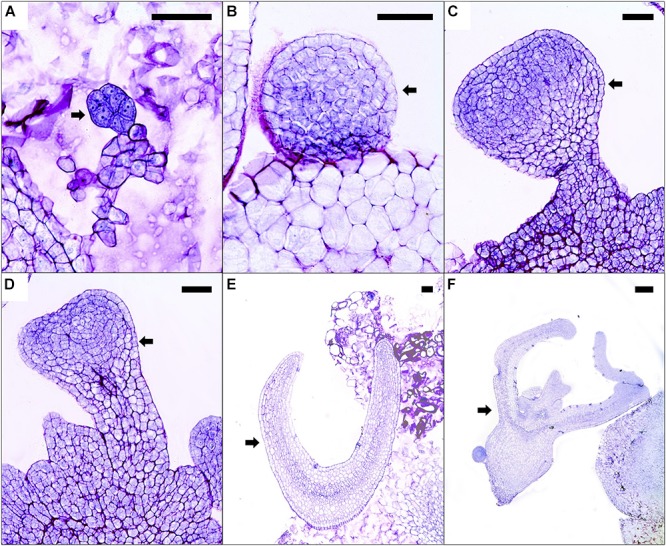
Histological sections of calluses with embryogenic structures cultured on medium supplemented with 9.0 μM 2,4-D after 8 months of culture. Arrows indicate a pro-embryo **(A)**, globular **(B)**, early-heart shaped **(C)**, heart-shaped **(D)**, torpedo-like **(E)**, and cotyledonary somatic embryos **(F)**. Black bars scale equivalent to 100 μm **(A–E)** and 200 μm **(F)**.

Somatic embryos developed on the embryogenic callus in a desynchronized way in all 2,4-D concentrations evaluated. Histological analysis of embryogenic calluses showed the presence of pro-embryos, characterized by their less-vacuolated cells with a denser cytoplasm and well-defined nucleuses in comparison with the cells in the surroundings (Figure 2A). Moreover, globular somatic embryos were found with a defined protodermis and without a suspensor-like structure (Figure 2B). From the former, the somatic embryos continued to differentiate into heart-shaped somatic embryos (Figures 2C,D). Further progression in the development of these proembryogenic structures was evident by the presence of somatic embryos in more advanced stages [e.g., torpedo-like structures and cotyledonary stage somatic embryos with normal anatomy (presence of radicle, hypocotyl, and cotyledons), Figures 2E,F]. No vascular connection between the somatic embryos, at any of the developmental stages, with the rest of the callus was observed (Figure 2).

### Multiplication and Germination of Papaya Somatic Embryos

**FIGURE 3:**
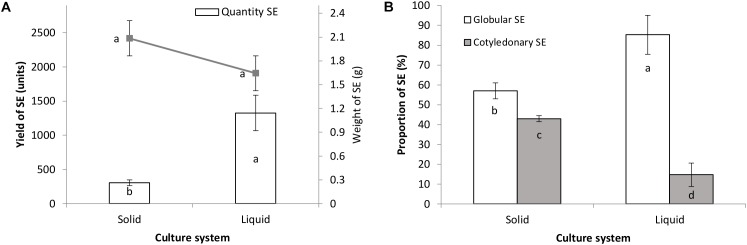
Multiplication of hybrid papaya somatic embryos and embryogenic callus growth **(A)** and the proportion of globular/cotyledonary SE formed **(B)** in liquid and semi-solid culture systems after a 2-month culture period. Statistical differences between treatments are shown as letters (*p* < 0.05). SE, Somatic embryos.

As can be seen in Figure 3A, after 8 weeks of culture the number of somatic embryos was significantly higher in the liquid culture system (1326 somatic embryos) compared with the semi-solid system (307 somatic embryos). Calluses with embryogenic structures in both culture systems showed a similar increase in fresh mass (Figure 3A). The frequency of globular somatic embryos was higher than that of the cotyledonary ones in the two systems evaluated; however, liquid cultures showed a significantly higher percentage of globular somatic embryos (85%) compared to the semi-solid cultures (57%), while the opposite occurred with the cotyledonary ones (Figure 3B).

**FIGURE 4:**
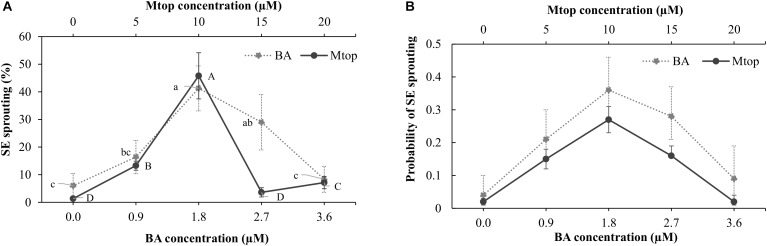
BA and Mtop concentration effect on the sprouting **(A)** of hybrid papaya somatic embryos after a 2-month culture and the sprouting probability based on a logistic regression model **(B)** for each plant growth regulator (BA: Wald *t*-test: *X*^2^ = 21.2, *p*(>*X*^2^) = 4.1e^-06^. Model equation: P(y) = 1/(1 + e^(3.0765-2.6089x+0.6763x^2^)^); *p-*value = 5.268642e^-07^ and Mtop: Wald *t*-test: *X*^2^ = 141.6, *p*(>*X*^2^) = 0.00. Model equation: P(y) = 1/(1 + e^(3.849376-0.562119x+0.027749x^2^)^); *p-*value = 5.268642e^-07^). Vertical bars correspond to the standard deviation of the means (A, B, and D) or of the calculated probabilities. Upper and lower cases denote significant differences between Mtop and BA concentrations, respectively (*p* < 0.05).

The addition of BA to the culture medium induced, after 8 weeks of culture, an increase in sprouting of papaya somatic embryos in concentrations of up to 1.8 μM, surpassing the 40% of sprouted somatic embryos. Higher concentrations (3.6 μM BA) showed a sprouting response similar to that of the control (Figure 4A). After a similar culture period, Mtop concentrations of up to 10 μM improved the sprouting of the somatic embryos, showing a 44% increase compared to the control. Higher concentrations of this compound did not induce somatic embryo sprouting (Figure 4A). Exogenous supplementation of BA or Mtop, as well, showed a significant interaction [*X*^2^ = 21.2, *p*(>*X*^2^) = 4.1e^-06^ and *X*^2^ = 141.6, *p*(>*X*^2^) = 0.0, respectively] with the sprouting response of the papaya somatic embryos. Despite the different levels of each plant growth regulator evaluated, a second-order polynomial trend was observed for each range of concentrations evaluated according to the modeling of the sprouting probability (*p* = 5.268642e^-07^ and 1.475542e^-44^, respectively) (Figure 4B).

**FIGURE 5:**
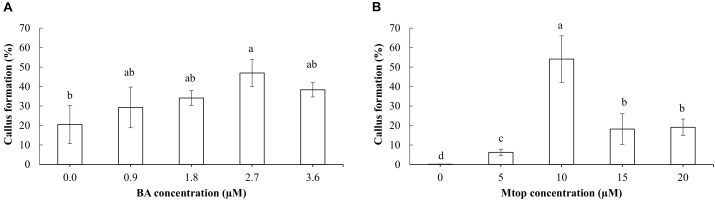
Effect of different concentrations of BA **(A)** and Mtop **(B)** on the formation of callus at the basal section of the hybrid papaya somatic embryos after 4 weeks of culture. Different letters show significant differences (*p* < 0.05) and bars indicate the standard error.

In addition, development of compact callus was observed in the basal zone of the somatic embryos, which ranged from 28 to 46% after 4 weeks of culture without significant differences according to the BA concentrations. Only somatic embryos cultured with 2.7 μM BA produced more of this callus type than the control (Figure 5A). In addition, 45% of the somatic embryos cultured on the medium supplemented with 3.55 μM BA showed oxidation symptoms, a value significantly higher (*p* < 0.05) to those obtained in the other treatments, which ranged between 13 and 23%, without significant differences among them (data not shown).

Addition of Mtop to the culture medium also induced callus formation in the basal zone of the corresponding somatic embryos. This callus was more friable than the one produced with BA and had a trichome-like form. An intermediate concentration of Mtop (10 μM) induced the highest callus formation in the somatic embryos compared to the other concentrations used (Figure 5B). Oxidation of somatic embryos ranged from 0 to 6% without significant differences (*p* > 0.05) among Mtop treatments (data not shown).

**FIGURE 6:**
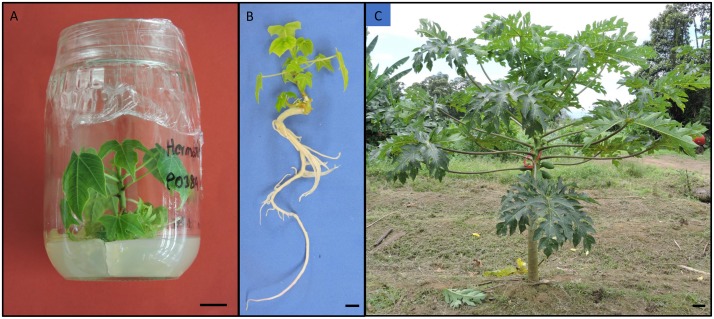
*In vitro* papaya plant ready for *ex vitro* acclimatization with 3–5 cm long **(A)**, a well-developed root system **(B)** and a field established plant with fruits and flowers **(C)** after 6 months of culture in Guapiles, Costa Rica, a humid tropical zone. Bars equivalent to 1 cm **(A,B)** and 10 cm **(C)**.

Radicle protrusion was observed in less than 5% of the somatic embryos cultured with BA, without significant differences among concentrations (data not shown, *p* > 0.05). Root development in those somatic embryos was characterized by the normal growth of the tap-root and the eventual formation of lateral roots (Figure 6B). Mtop did not induce rooting in any concentration tested. During acclimatization, survival of rooted plantlets measuring approximately 5 cm long (Figures 6A,B) reached 60% after 3 months. The acclimatized plants sowed in the field showed normal growth and were able to flower and set fruit (Figure 6C) 4 months after transplanting.

### Encapsulation of Papaya Somatic Embryos

**FIGURE 7:**
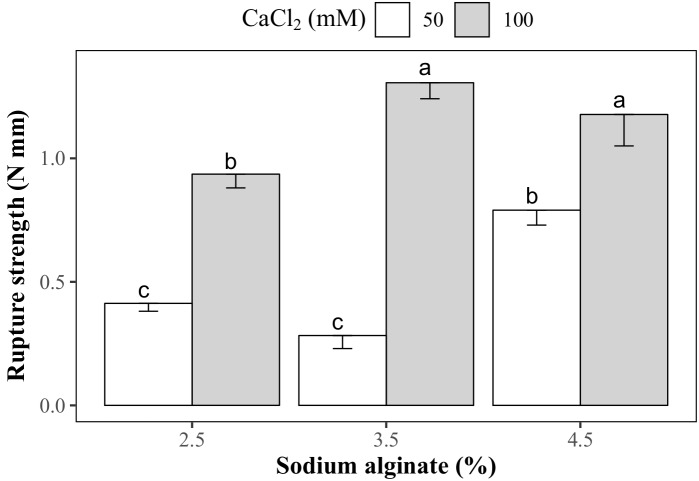
Hardness of the calcium alginate capsules prepared with different sodium alginate and CaCl_2_ concentrations.

**FIGURE 8:**
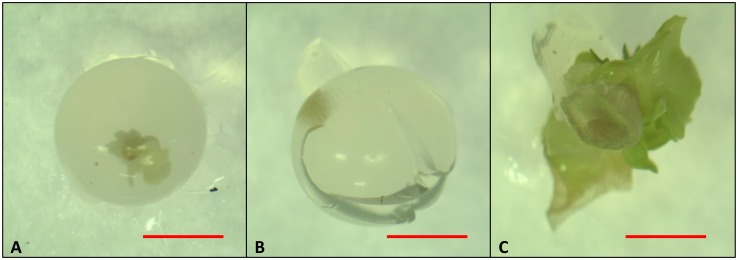
Papaya artificial seeds (4.5% sodium alginate and 100 mM CaCl_2_) with or without a treatment with 200 mM de KNO_3_ for 20 min (**A,B**, respectively), and sprouted seed after 12 weeks of culture **(C)**. Bars equal 3 mm.

Droplets with 3.5 and 4.5% sodium alginate and 100 mM CaCl_2_ were the hardest, with rupture strength values of 1.31 and 1.18 N mm, respectively (Figure 7). Calcium alginate capsules made with 2.5 and 3.5% sodium alginate and 50 mM CaCl_2_ solutions had the lowest rupture strength (0.41 and 0.28 N mm, respectively). Intermediate rupture strength values were obtained in the capsules produced with 2.5 and 4.5% sodium alginate and 50 and 100 mM CaCl_2_ solutions, respectively. Rupture strength of the KNO_3_-treated capsules could not be determined since they cracked after immersion in this salt solution (Figure 8A). Even though the concentration of both encapsulating solutions (sodium alginate and CaCl_2_) affected the hardness of the capsules, no significant correlation was found between this variable and the sprouting percentage of the encapsulated somatic embryos (correlation coefficient: 0.02).

The sprouting percentage of the encapsulated somatic embryos after 1 month of culture was between 4 and 10% for all the encapsulation treatments (*p* > 0.05) (data not shown). Non-encapsulated embryos sprouted at significantly higher rates (30%). Figures 8B,C show an encapsulated somatic embryo before and after a 3-month culture period.

## Discussion

### Formation and Characterization of Callus

Development of non-embryogenic callus in the hypocotyl segments was observed after 8 weeks of culture in more than 90% of the explants of the evaluated hybrid regardless of the 2,4-D or sucrose concentrations used. Fitch (1993) reported callus formation in hypocotyl segments of several papaya genotypes cultured with 2,4-D as soon as 2 weeks after culture started. Moreover, after 10–14 weeks at least 10% of the calluses of three genotypes acquired embryogenic competence, a response not observed in hypocotyls of our hybrid even beyond the mentioned culture period. Response of explants to particular tissue culture conditions is highly dependent of genetic and physiological determination (Jiménez, 2005; Fehér, 2006). In the particular case of papaya, differential responses of hypocotyl segments of the “Kapoho,” “Sunset,” “Sunrise,” “Waimanalo,” and “Tainung” genotypes using different concentrations of 2,4-D (2.3–112.5 μM) have been documented, showing a positive response in the first four Hawaiian genotypes in comparison with “Tainung,” a Taiwanese genotype in which no somatic embryos were obtained (Fitch, 1993; Almeida et al., 2001). This genetic determination could be partially explained by a differential regulation of transcription factors through the modification of the DNA methylation status between cultivars, such as has been reported for *Pinus radiata* cultivars with contrasting embryogenic competence (Bravo et al., 2017). In addition, papaya polar auxin transport genes (*AUX1/LAX* and *PIN* family genes) are expressed since the initial stages of somatic embryogenesis (Estrella-Maldonado et al., 2018), and their regulation could affect the initial polarization of the callus cells thus promoting or hampering induction of the embryogenic process.

Regarding the half-cut seeds, formation of callus with embryogenic structures after 2 months was similar in the explants cultured in a 2,4-D range from 9.0 to 27.1 μM. Analogous results were obtained when inducing this type of callus in “CO7” cultivar using the same 2,4-D concentrations, and likewise, higher values of this growth-regulator reduced the percentage of callus formation (Anandan et al., 2012). Differentiation of somatic embryos in the hybrid used in the present work occurred indirectly from cellular callus clusters (Figure 2); a similar pattern has been reported in other papaya cultivars (Vale et al., 2018) as well as in *Passiflora ligularis* (Prudente et al., 2017).

Sucrose concentration did not influence the development of callus with embryogenic structures on our half-cut seeds. In a previous report, higher sucrose concentration increased the development of somatic embryos in callus from Hawaiian genotypes, which could be related to the osmotic potential of the culture medium (Fitch, 1993). Supporting this, an increase in the number of somatic embryos was observed in the papaya hybrid UENF/CALIMAN01 when calluses, formed from immature zygotic embryos, were treated with polyethylene glycol (60 g l^-1^) for 1 month (Vale et al., 2014). More recently, Vale et al. (2018) induced callus with embryogenic structures in the aforementioned cultivar on MS culture medium supplemented with 30 g l^-1^ sucrose and 20 μM 2,4-D and improved the maturation of the somatic embryos by inducing an osmotic stress through the addition of 60 g l^-1^ sucrose to the culture medium.

Contrasting to the results of the present work, induction of callus with embryogenic structures has been reported with isolated zygotic embryos in several papaya cultivars (“CO7,” “Solo,” “Maradol,” and “Maradol Roja”) (Fitch and Manshardt, 1990; Castillo et al., 1998; Posada-Pérez et al., 2007; Ascencio-Cabral et al., 2008; Anandan et al., 2012). In addition to genetic considerations, this behavior could be related to the physiological maturity-state of the seeds used [approximately 130–150 days after anthesis (daa) in the present work]. Anandan et al. (2012) observed a null response to 2,4-D inductive treatments in “CO7” cultivar seeds harvested 140–150 daa, but seeds collected at 110–120 daa showed a positive response.

### Multiplication and Germination of Papaya Somatic Embryos

The largest number of papaya somatic embryos obtained by subculturing the embryogenic calluses in liquid culture medium than on semi-solid can be related to the advantages offered by the former culture system. Better cluster dissociation, greater exposure of tissues to the culture environment and its components and good ventilation, caused by Erlenmeyer flasks agitation, are benefits of the liquid culture and are considered to have a huge influence on the formation of new somatic embryos (Jiménez, 2001; Remakanthan et al., 2013). Similar results were observed comparing liquid and semi-solid culture systems for the embryogenic callus multiplication of *Phoenix dactylifera* cultivars “Deglet Nour,” “Bousthami noir,” and “Jihel,” obtaining in some cases a 20% increase in the number of somatic embryos in the liquid culture (Fki et al., 2003; Al-Khayri, 2012). Moreover, Litz and Conover (1983) succeeded in establishing papaya embryogenic suspensions in a MS culture medium supplemented with 2,4-D (9.0 μM), after a similar culture period than the one used in this study (1–2 months). Lastly, calluses with embryogenic structures showed the same growth in both culture systems.

The greatest number of somatic embryos in the globular developmental stage obtained in the liquid system indicates an effect of the culture system on the development of newly formed somatic embryos. It is considered that the differentiation of globular embryos to later stages of embryonic development is inhibited by continuous culture in the presence of auxins, due to the synthesis of mRNA and proteins that stop embryonic development (Jiménez, 2001). Moreover, liquid culturing may have shifted the genome methylation patterns of our papaya somatic embryos, changing the expression of key developmental genes. In *Acca sellowiana* somatic embryos, the exogenous application of 5-azacytidine (10 μM), a demethylating agent, with 2,4-D (200 mM) caused hypomethylation, stopping their development before reaching the heart stage (Fraga et al., 2012).

BA has been used in many species to promote germination of somatic embryos (Raemakers et al., 1995; Fehér, 2006). Optimal range of BA concentration for the sprouting of our papaya somatic embryos was between 0.9 and 2.7 μM. This result is in agreement with Posada-Pérez et al. (2007, 2017), who observed 92% germination of “Maradol Roja” somatic embryos using BA (∼0.9 μM) after 30 days of culture.

Moreover, sprouting probability was higher (∼0.25) in the somatic embryos of this work cultured with 10 μM Mtop compared to the rest of the concentrations evaluated. This aromatic cytokinin has been used to promote maturation of *Albizia lebbeck* somatic embryos at 7.5 μM and it has shown a reduction in the number of abnormal shoots of *Barleria greenii* compared to equimolar concentrations of BA (Amoo et al., 2010; Saeed and Shahzad, 2015). This is the first work known to the authors about the use of Mtop in papaya.

Somatic embryos of our papaya hybrid, indistinctively of the concentration and the growth regulator used (BA or Mtop), showed very low rooting capability. Clarindo et al. (2008) obtained similar results cultivating “Golden” papaya somatic embryos on MS medium devoid of growth regulators. This behavior could be related to the final development and maturity stage reached by the somatic embryos after their subculture under germination conditions. Vale et al. (2018) reported that maturating papaya somatic embryos with polyethylene glycol (60 g l^-1^) reduced the quantity of abnormal embryos and promoted their complete conversion into bipolar structures. This maturation process induced by polyethylene glycol is characterized by an increase in the internal sucrose and starch contents as well as with a differential protein profile than in the non-treated somatic embryos (Vale et al., 2014, 2018). Moreover, Pullman et al. (2003) observed that cotyledonary and white zygotic embryos of *Pinus taeda* classified as mature had suitable germination (e.g., growth of the radicle first and then of the hypocotyl), but, cotyledonary embryos at an earlier developmental stage did not show any root growth and the hypocotyl developed first.

The low germination rate of our papaya somatic embryos observed in the culture medium without growth regulators probably indicates the necessity of applying an additional stimulus to promote germination. Low complete germination of somatic embryos of the Cuban cultivar “Maradol Roja” has been overcome by implementing temporary immersion systems (RITA^®^) with half-strength MS medium containing 0.02 μM BA, 2.90 μM gibberellic acid and an optimal inoculum density of 200 mg of fresh somatic embryos or supplementing the semi-solid culture medium with 475.8 μM phloroglucinol (1,3,5-trihydroxy benzene), obtaining 95 and 100% of somatic embryos with radicle and apical growth, respectively (Posada-Pérez et al., 2017). These approaches could be an alternative to the protocol described in this study to increase the number of somatic embryos with complete germination.

### Encapsulation of Papaya Somatic Embryos

Synthetic seeds in this work showed reduced sprouting when compared to non-encapsulated somatic embryos regardless of the sodium alginate and CaCl_2_ concentrations used. Singh and Chand (2010) observed a similar response in *Dalbergia sissoo*, where the encapsulation of the synthetic seeds (sodium alginate 2.5% and 75 mM CaCl_2_) reduced the sprouting of somatic embryos down to 29%. The encapsulation matrix, besides being a physical barrier, also modifies the water potential in the surroundings of the somatic embryos due to an increase in the concentration of sodium ions, affecting water availability for the somatic embryos (West et al., 2006). This could be an explanation for the negative effect exerted by encapsulation, regardless of the concentration of sodium alginate or CaCl_2_, on papaya somatic embryos sprouting. Castillo et al. (1998) reported 80% germination of papaya synthetic seeds produced with 2.5% sodium alginate in a 50 mM CaCl_2_ solution, but the exposure time on this last solution was of 10 min, much less than the 30 min used in this work. However, Castillo et al. (1998) did not measure the rupture strength of the capsules.

The expected relationship between the concentration of sodium alginate and of CaCl_2_ over the rupture strength of the calcium alginate capsules is also consistent with the results obtained by Timbert et al. (1995), who observed a higher rupture strength while increasing the sodium alginate (0.75–2.5%) and CaCl_2_ (25–100 mM) concentrations in the synthetic seeds of *Daucus carota* L. In spite of the former, no significant interaction was found between the hardness of the calcium alginate capsules and the rate of papaya somatic embryo sprouting in this work, even when the synthetic seeds were treated with KNO_3_ to soften the capsule. According to Timbert et al. (1995), a high correlation between the rupture strength of the beads, regulated by the concentration of sodium alginate and CaCl_2_, and the germination of the encapsulated somatic embryos of *D. carota* is obtained when high viscosity (250 mPa s) sodium alginate is used. In the present study, the sodium alginate used had a viscosity of 80 mPa s which might have affected the correlation between the hardness of the capsules and germination of our somatic embryos.

Summarizing, in the present work it was not possible to induce embryogenic competence neither in hypocotyls nor in isolated zygotic embryos of the papaya hybrid used. Callus with embryogenic structures was only observed in half-cut seeds with up to 27.1 μM 2,4-D, while higher concentrations tended to reduce it. Moreover, sucrose concentration did not affect formation of somatic embryos. Liquid culture induced a higher rate of somatic embryo multiplication and of globular/cotyledonary somatic embryos compared with the semi-solid culture. BA and Mtop promote the sprouting of somatic embryos up to concentrations of 2.7 and 10 mM, respectively, but root formation was observed only in BA treatments. Moreover, acclimatized plantlets from the germinated (bipolar) somatic embryos developed normally in the field, setting flowers and fruits. Encapsulation reduced the sprouting response in all sodium alginate and CaCl_2_ concentrations used, however, without any apparent relationship to the rupture strength of the beads.

## Author Contributions

PS-C, NS-C, and VMJ designed the experiments. NS-C and PS-C conducted the experiments. PS-C analyzed the data. PS-C and VMJ wrote the manuscript. All authors reviewed and approved the manuscript.

## Conflict of Interest Statement

The authors declare that the research was conducted in the absence of any commercial or financial relationships that could be construed as a potential conflict of interest.
